# Editorial: Clinical application of artificial intelligence in emergency and critical care medicine, volume III

**DOI:** 10.3389/fmed.2022.1075023

**Published:** 2022-12-19

**Authors:** Zhongheng Zhang, Rahul Kashyap, Longxiang Su, Qinghe Meng

**Affiliations:** ^1^Department of Emergency Medicine, Key Laboratory of Precision Medicine in Diagnosis and Monitoring Research of Zhejiang Province, Sir Run Run Shaw Hospital, Zhejiang University School of Medicine, Hangzhou, China; ^2^Key Laboratory of Digital Technology in Medical Diagnostics of Zhejiang Province, Hangzhou, China; ^3^Critical Care Independent Multidisciplinary Program, Mayo Clinic, Rochester, MN, United States; ^4^Department of Anesthesiology and Perioperative Medicine, Mayo Clinic, Rochester, MN, United States; ^5^State Key Laboratory of Complex Severe and Rare Diseases, Department of Critical Care Medicine, Peking Union Medical College Hospital, Chinese Academy of Medical Science and Peking Union Medical College, Beijing, China; ^6^Department of Surgery, State University of New York Upstate Medical University, Syracuse, NY, United States

**Keywords:** artificial intelligence, critical care, heterogeneity, prediction, diagnosis

Two years have passed since the first launch of the Research Topic on the application of artificial intelligence (AI) in emergency and critical care settings (1). We have witnessed increasing submissions to this topic over these years, indicating continued research interest among the critical care community. AI is a data analysis approach that has revolutionized many industry areas (2, 3), as well as clinical medicine (4). With more data being captured and stored during routine clinical practice, the large volumes of data have the potential to reveal more knowledge to better inform clinical decision makings (5). In general, clinical questions involving all stages of clinical practice including diagnosis, treatment, and prognosis can be well-investigated by the employment of AI technology. Figure 1 illustrates how AI can help to make better patient care in all stages of clinical practice. Diagnosis is usually the first step in the management of patients. Prompt and accurate diagnosis can help better patient treatment in the critical care setting. For instance, there has been a large body of evidence showing that early initiation of a sepsis care bundle can help to improve survival outcomes, and thus strenuous efforts have been made to provide early warning for sepsis. The automated early warning system has been widely applied in the clinical setting, and preliminary studies show promising results (6). With the help of AI, the identification of sepsis can be done earlier with increased accuracy (7). The second aspect relating to the diagnosis refers to the sub-classification of a heterogeneous syndrome. Many diseases or syndromes in the critical care setting encompass a heterogenous population and the identification of subtypes can help tailor treatment strategies (8). In volume III of the topic series, Wu et al. trained a classification model on facial expressions video clips, and their deep learning method is shown to accurately classify patients with or without pain. This important study implies that pain assessment can be achieved by an automated computer system, thereby providing high granularity time-varying facial expression data for patient management. Sepsis-Associated Thrombocytopenia (SAT) is an important complication in sepsis patients and early risk stratification can help to tailor individualized treatment. Jiang et al. trained multiple machine learning (ML) models for the prediction of SAT in a Chinese cohort, and then these models were validated in an open-access critical care database.

**Figure 1:**
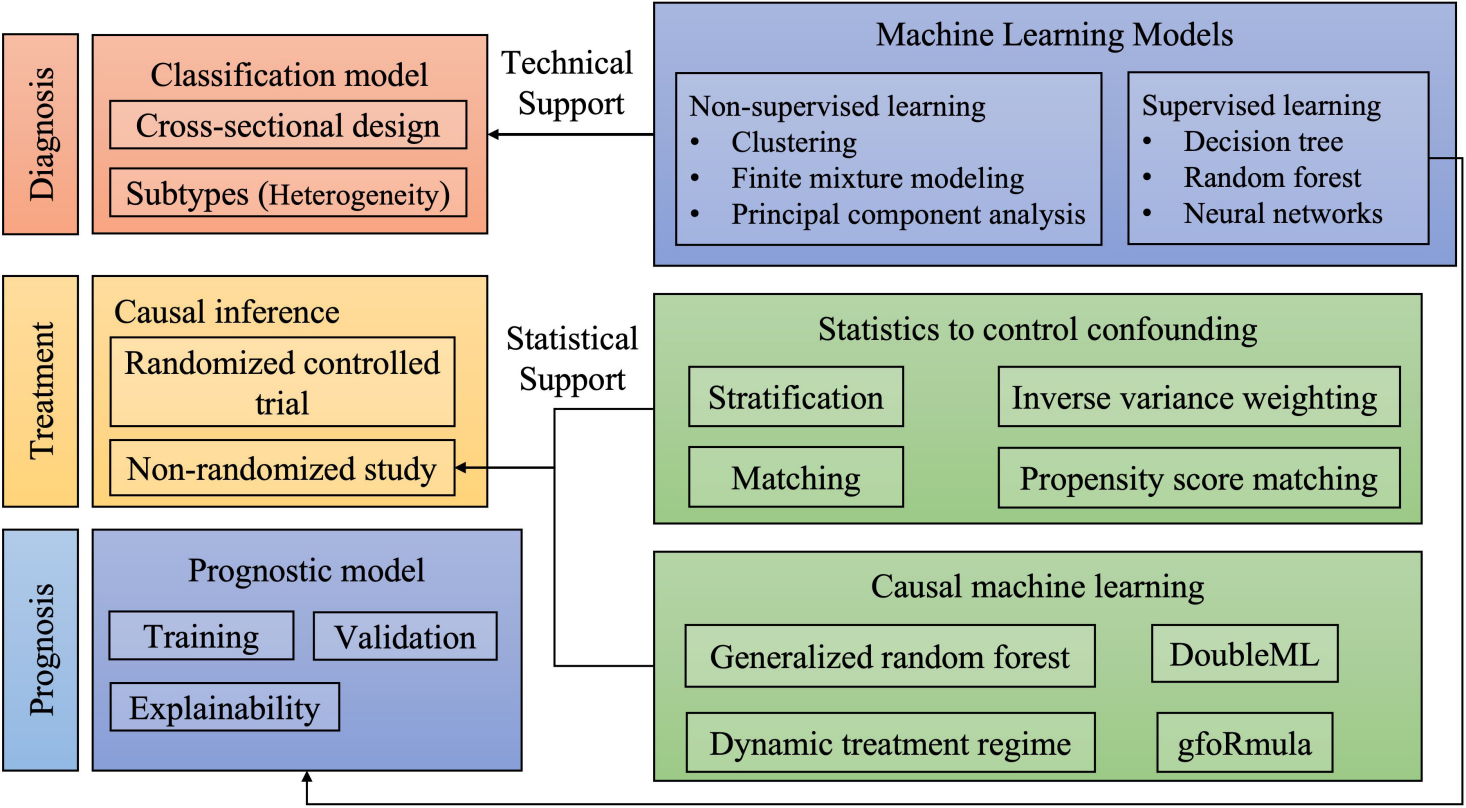
Illustrations of the application of artificial intelligence to various aspects of clinical practice.

The second step in patient management involves the treatment strategy. Since critically ill patients are usually treated with multi-module strategies, the effectiveness of treatment strategies is time sensitive, and varies across the individual subject. Thus, an individualized treatment strategy is needed, in line with the idea of precision medicine. Mechanical ventilator (MV) weaning is an important medical decision-making process for the management of patients on MV. Liu et al. developed an AI algorithm to dictate MV weaning. This study paves the way for the realization of personalized medicine in the management of MV patients.

Finally, the prognosis is also important for the management of critically ill patients. Risk stratification for intensive care unit (ICU) patients is useful for clinicians to make better decisions and for consulting with family members. The diagnosis and prognosis can be studied with the supervised ML algorithm. The difference lies in the study design. While the studies on diagnostic performance require cross-sectional data to train the model, those involving prognostic performance require a follow-up period allowing the outcome (label) to occur. ICU readmission is an important indicator of the quality of care and is an important outcome measurement. In this topic issue, Hegselmann et al. developed an explainable boosting machine to predict ICU re-admission using a German dataset.

In conclusion, the successful launch of the special issue on the application of AI in critical care medicine indicates that researchers continue to be interested in this particular field. The power of big data and AI are revolutionizing clinical practice in the near future. The ICU is a highly technological environment where each patient generates a large volume of data per day, such special characteristics make it the best place for AI applications.

## Author contributions

ZZ conceived the idea and drafted the manuscript. LS and QM revised the paper. RK worked to organize the topic issue and made contributions to the editorial contents. All authors contributed to the article and approved the submitted version.
